# AIDS-Related Tuberculosis in Rio de Janeiro, Brazil

**DOI:** 10.1371/journal.pone.0003132

**Published:** 2008-09-10

**Authors:** Antonio G. Pacheco, Betina Durovni, Solange C. Cavalcante, L. M. Lauria, Richard D. Moore, Lawrence H. Moulton, Richard E. Chaisson, Jonathan E. Golub

**Affiliations:** 1 ENSP/PROCC, FIOCRUZ, Rio de Janeiro, Brazil; 2 IPEC, FIOCRUZ, Rio de Janeiro, Brazil; 3 Municipal Health Secretariat, Rio de Janeiro City, Brazil; 4 Federal University of Rio de Janeiro, Rio de Janeiro, Brazil; 5 Center for Tuberculosis Research, Johns Hopkins University, Baltimore, Maryland, United States of America; 6 Department of Medicine, Johns Hopkins University, Baltimore, Maryland, United States of America; 7 Department of Epidemiology, Johns Hopkins University, Baltimore, Maryland, United States of America; 8 Department of International Health, Johns Hopkins University, Baltimore, Maryland, United States of America; Harvard Medical School, United States of America

## Abstract

**Background:**

We studied the incidence of tuberculosis, AIDS, AIDS deaths and AIDS-TB co-infection at the population level in Rio de Janeiro, Brazil where universal and free access to combination antiretroviral therapy has been available since 1997.

**Methodology/Principal Findings:**

This was a retrospective surveillance database match of Rio de Janeiro databases from 1995–2004. Proportions of tuberculosis occurring within 30 days and between 30 days and 1 year after AIDS diagnosis were determined. Generalized additive models fitted with cubic splines with appropriate estimating methods were used to describe rates and proportions over time. Overall, 90,806 tuberculosis cases and 16,891 AIDS cases were reported; 3,125 tuberculosis cases within 1 year of AIDS diagnosis were detected. Tuberculosis notification rates decreased after 1997 from a fitted rate (fR per 100,000) of 166.5 to 138.8 in 2004. AIDS incidence rates increased 26% between 1995 and 1998 (30.7 to 38.7) followed by a 33.3% decrease to 25.8 in 2004. AIDS mortality rates decreased dramatically after antiretroviral therapy was introduced between 1995 (27.5) and 1999 (13.4). The fitted proportion (fP) of patients with tuberculosis diagnosed within one year of AIDS decreased from 1995 (24.4%) to1998 (15.2%), remaining stable since. Seventy-five percent of tuberculosis diagnoses after an AIDS diagnosis occurred within 30 days of AIDS diagnosis.

**Conclusions/Significance:**

Our results suggest that while combination ART should be considered an essential component of the response to the HIV and HIV/tuberculosis epidemics, it may not be sufficient alone to prevent progression from latent TB to active disease among HIV-infected populations. When tuberculosis is diagnosed prior to or at the same time as AIDS and ART has not yet been initiated, then ART is ineffective as a tuberculosis prevention strategy for these patients. Earlier HIV/AIDS diagnosis and ART initiation may reduce TB incidence in HIV/AIDS patients. More specific interventions will be required if HIV-related tuberculosis incidence is to continue to decline.

## Introduction

The impact of the human immunodeficiency virus/acquired immune deficiency syndrome (HIV/AIDS) epidemic on tuberculosis (TB) incidence has been catastrophic, underscoring the need for new approaches to controlling these overlapping diseases [1], [2]. Cohort studies [3]–[10] have shown that combination antiretroviral therapy (ART) reduces the risk of active tuberculosis in individuals compared to no treatment, but the ecological level impact of ART on tuberculosis rates is not yet known.

In Brazil, antiretrovirals have been available free of charge for HIV patients since 1991 and combination therapy including protease inhibitors since 1997. Rio de Janeiro presents a unique setting to evaluate the population level impact of ART on the incidence of HIV-related tuberculosis. If combination ART significantly reduces active tuberculosis incidence among populations with HIV/AIDS, then the global scale up of HIV therapy could contribute to improved tuberculosis control. Conversely, the lack of an appreciable effect would suggest that alternative strategies are needed.

We studied the incidence of active tuberculosis, AIDS, AIDS deaths and tuberculosis with AIDS in the same year in Rio de Janeiro from 1995–2004 using surveillance databases maintained by the City Health Secretariat. Reporting of tuberculosis and AIDS is mandatory, and both ART and anti-tuberculosis drugs are distributed exclusively by the government. By measuring rates of both tuberculosis and AIDS in the population of an entire large city, we would be able to assess trends in AIDS-related tuberculosis in the era of combination antiretroviral therapy.

## Methods

Three official databases (described below) of the City Health Secretariat of Rio de Janeiro were used to determine total number of AIDS cases, tuberculosis cases, AIDS deaths and AIDS/tuberculosis cases between 1995 and 2004, and the annual incidences of each. The official databases of AIDS and tuberculosis cases reported to the Health Department (The Sistema de Informação de Agravos de Notificação – AIDS and Tuberculosis [SINAN – AIDS and SINAN-TB] and the Sistema de Informação sobre Mortalidade [SIM]) are Brazilian nation-wide mandatory reporting systems for AIDS, tuberculosis, and deaths, respectively. The AIDS data have been maintained in an electronic database in the City Health Department since the first AIDS case was reported in 1982, as has been the situation for tuberculosis cases reported since 1995 and for deaths since 1979. Patients are included in databases when they are diagnosed with either AIDS or tuberculosis according to the Brazilian guidelines at the time of diagnosis. Information collected includes demographic and specific clinical information, including name, date of birth, mother's name and date of diagnosis. The forms are completed by a health professional in a primary care unit or in a hospital on the day the individual is diagnosed with the disease. The forms are then sent to the City Health Department and entered into a main electronic database, developed by the Brazilian Ministry of Health. Information on mortality is also kept in an electronic database and is based on the death certificate, completed by the attending physician at the time of death. In Brazil, tuberculosis is diagnosed in patients presenting with signs and symptoms compatible with tuberculosis on the basis of chest radiographs, sputum acid-fast bacilli smears, and response to anti-tuberculosis therapy [11], [12]. An AIDS diagnosis is based on clinical and/or immunological criteria as in the WHO definition [13]. However, tuberculosis is not necessarily an AIDS defining illness in HIV-infected persons in Brazil, but contributes a score of 5 in a 10 point scale required for an AIDS diagnosis [14].

In order to identify tuberculosis cases among AIDS patients, both the information about tuberculosis at the time of notification and a database linkage between AIDS and tuberculosis databases was performed. The primary components of the matching algorithm were patient name (last and first), mother's name, and date-of-birth. Briefly, a hierarchical algorithm combining an adaptation of the Soundex phonetic algorithm to the Portuguese language and a similarity score for strings implemented in the library difflib from Python [15] was used to determine the degree of similarity between records in the databases. Pairs of records were classified as a true match, a non-match, and, when the algorithm was not able to determine the status automatically, records were checked manually. Preliminary results of the linkage algorithm, performed with patients in two large HIV-infected cohorts in Rio de Janeiro and the mortality database, indicated that the algorithm has high sensitivity (∼92%) and specificity (∼99%) to detect true matches. The algorithm was written in Python for Windows version 2.4 [15].

Patients were considered to have tuberculosis at the same time as their AIDS diagnosis if they either had tuberculosis mentioned in their AIDS report form or if they had a tuberculosis episode found through linkage up to 30 days before AIDS diagnosis. In another definition, we also included those who had tuberculosis up to 30 days after AIDS diagnosis as having tuberculosis at AIDS diagnosis. These definitions were necessary because the surveillance systems are separate and so are physicians who report AIDS and/or tuberculosis, even in the same heath care facility, and small delays in reporting one or the other condition is expected. The two definitions were combined to provide the number of simultaneous diagnoses.

### Analysis

To determine the proportion of AIDS patients who were diagnosed with tuberculosis within one year following their AIDS diagnosis, annual cohorts of patients diagnosed with AIDS in a calendar year were defined as denominators with tuberculosis diagnosed within up to one year since AIDS diagnosis among this population as numerators. Municipal surveillance estimates of number of AIDS patients receiving combination ART have been used to overlay graphs generated for this analysis. Generalized additive models (GAM) [16] were used to adjust the incidences and proportions along with 95% confidence intervals (CI). All models used cubic splines with 3 knots and quasipoisson and quasibinomial families were used for rates and proportions, respectively, to account for within-year correlation. Proportions of AIDS-TB cases over time were calculated from 1995 and 2003 only, in order to allow 1 year to accrue tuberculosis cases among them. Separate analyses were carried out for those who had tuberculosis within 30 days from AIDS diagnosis (considered as tuberculosis at AIDS diagnosis) and those who had it between 30 days and one year. These separate analyses were conducted to describe trends in prevalent tuberculosis disease (simultaneous diagnoses) and incident disease (diagnosed between 30 days and one year following AIDS diagnosis). All analyses were done with R for Windows version 2.4.1 [17].

## Results

Between 1995 and 2004, 90,806 tuberculosis cases and 16,891 AIDS cases were reported in Rio de Janeiro. Initially, the AIDS registry listed 2,160 patients with a concurrent tuberculosis diagnosis. The 90,806 patients recorded in the tuberculosis registry were merged with 14,371 AIDS patients who did not have tuberculosis reported at AIDS diagnosis. This merge yielded an additional 1,962 coinfected cases. After comparing the registries and removing duplicates and recurrences, we found 3,125 AIDS cases who had one tuberculosis episode within one year of AIDS reporting, with 2,436 diagnosed within the first 30 days and 689 between 30 days and one year. The 3,125 tuberculosis episodes within one year of AIDS notification between 1995 and 2003 constituted 3.45% of all tuberculosis patients and 18.5% of all AIDS patients during that time.

**Figure 1:**
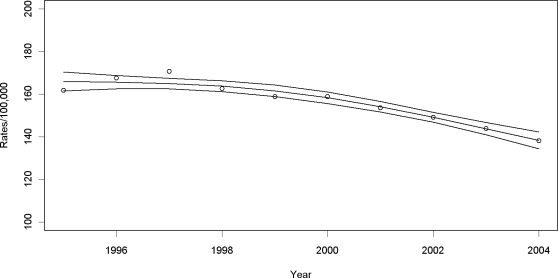
Tuberculosis notification rates, fitted rates and 95% confidence intervals in Rio de Janeiro between 1995 and 2004.

**Figure 2:**
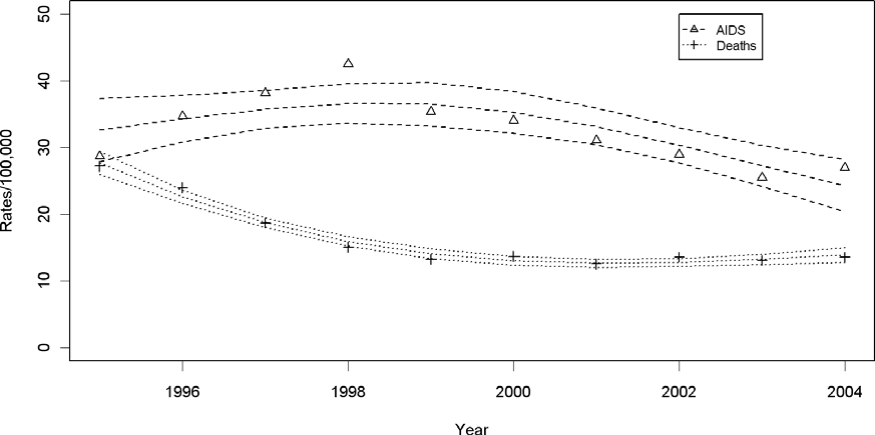
AIDS incidence rates (open triangles) and AIDS death rates (crosses) with fitted rates and 95% confidence intervals in Rio de Janeiro between 1995 and 2004.

Tuberculosis notification rates (Figure 1) had a slight increase from 1995–1997, and then decreased 16.6% between 1997 and 2004 from a fitted rate of 166.5 per 100,000 person-years (95%CI = 161.6, 167.2) to a fitted rate of 138.8 (95%CI = 135.1, 142.6). Figure 2 displays the increasing AIDS incidence rates between 1995 and 1998 (26% in fitted rates from 30.7 to 38.7) followed by a 33.3% decrease to 25.8 (95%CI = 22.9, 28.7) in 2004. Figure 2 also illustrates the dramatic reduction in AIDS mortality after ART was introduced from 1,537 deaths (fitted rate = 27.5; 95%CI = 25.7,29.2) in 1995 to 769 deaths (fitted rate = 13.4; 95%CI = 12.3,14.6) in 1999.

**Figure 3:**
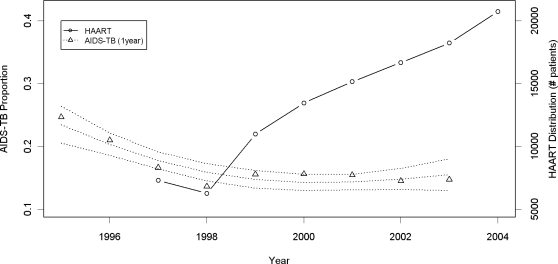
Proportions of AIDS-TB within one year of AIDS diagnosis (open triangles) with fitted proportions and 95% confidence intervals between 1995 and 2003 and number of patients receiving combination ART (open circles) between 1995 and 2004 in Rio de Janeiro.

The proportion of patients with tuberculosis diagnosed within one year of AIDS diagnosis (including those between 0 and 30 days) over this time period is shown in Figure 3, along with the number of patients receiving combined ART since 1997. The fitted proportion of AIDS cases who had tuberculosis within one year of AIDS diagnosis decreased from 24.4% in 1995 (95%CI = 22.1, 26.6) to 15.2% in 1998 (95%CI = 14.1, 16.3), remaining stable since then, reaching 14.7% in 2003 (95%CI = 12.9, 16.6). There has been no significant change in AIDS mortality rates since 1999 or tuberculosis proportion within 1 year of AIDS diagnosis since 1998, despite the threefold increase in combined ART (from 7,297 patients in 1997 to 20,749 patients in 2004).

**Figure 4:**
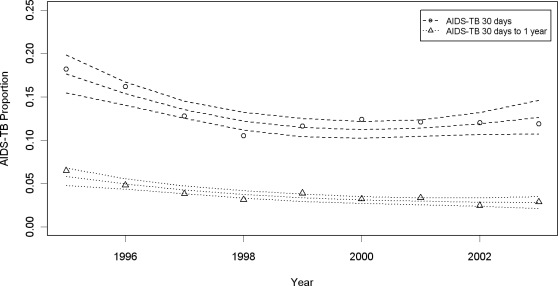
Proportion of AIDS-TB within 30 days of AIDS diagnosis (open circles) and between 30 days and one year (open triangles) with fitted proportions and 95% confidence intervals between 1995 and 2003 in Rio de Janeiro.

In Figure 4, the proportion of tuberculosis cases diagnosed within 30 days of AIDS diagnosis is shown (open circles) along with the proportion of tuberculosis cases diagnosed between 30 days and 1 year after AIDS diagnosis (open triangles). From these graphs, it is evident that the majority of coinfected patients (75%) are diagnosed with tuberculosis within 30 days of AIDS diagnosis. Proportions have steadily decreased among those diagnosed within 30 days of their AIDS diagnosis, from 18.2% in 1995 (95%CI = 16.5, 19.9) to 11.7% in 1998 (95%CI = 10.9, 12.5), remaining stable since then with a fitted proportion of 12.0% in 2003 (95%CI = 10.5, 13.5). The proportions of patients diagnosed with tuberculosis between 30 days and 1 year after their AIDS diagnosis also decreased from 6.2% (95%CI = 5.2, 7.2) in 1995 to 3.5% (95%CI = 3.1, 4.0) in 1998, with a continued slight decrease thereafter, reaching 2.7% in 2003 (95%CI = 2.1, 3.3).

## Discussion

The population burden of AIDS-related tuberculosis in Rio de Janeiro decreased significantly between 1995 and 1998, however little change occurred between 1998 and 2003 despite the widespread increased distribution of HAART for AIDS patients during this time. Active tuberculosis notification rates have been steadily decreasing in Rio de Janeiro as has overall AIDS incidence. AIDS mortality has also been stable since 1999 despite the increase in HAART distribution. Though the proportion of patients developing tuberculosis after AIDS declined between 1993 and 2002, the magnitude of decrease was substantially less than that seen with other opportunistic infections, such as pneumocystis pneumonia and toxoplasmosis [18] and the proportion of AIDS patients who were later diagnosed with tuberculosis remained stable. Moreover, the majority (75%) of active tuberculosis cases diagnosed among AIDS patients was diagnosed within 30 days of AIDS diagnosis and there was no significant reduction in the proportion of tuberculosis cases diagnosed within 30 days of an AIDS diagnosis after 1998. Though fewer tuberculosis cases were being diagnosed between 30 days and 1 year of an AIDS diagnosis, only moderate overall reductions in tuberculosis diagnoses were observed, even though a negligible decrease in the proportions was also observed after 1998.

Overall AIDS mortality in Rio de Janeiro fell by 50% from 1995 to 1999, remaining somewhat stable since then. Beginning in 1997, combined antiretroviral use in Rio de Janeiro increased substantially. The declining trend in mortality in Brazil before the widespread availability of HAART was well documented both at the individual level [19] and at the population level [20] and could be attributed to the universal availability of other therapeutic schemes, and the protection of ART on TB-AIDS co-infection must have followed the same trend. Thus, the most plausible explanation for not finding a reduction in tuberculosis after AIDS is that in Rio de Janeiro a high proportion of the population has latent tuberculosis and, therefore, tuberculosis occurs before HIV-infected. patients meet criteria for, or gain access to, ART. Another explanation would be delays in HIV diagnosis, leading patients to be diagnosed when they get tuberculosis diagnosed together with other criteria for AIDS definition. Our data shows that 75% of AIDS patients with active tuberculosis are actually being diagnosed with tuberculosis at the same time or soon after AIDS diagnosis when antiretroviral therapy would be initiated. Modeling suggests that ART will result in greater reductions in tuberculosis incidence if started at CD4 cell counts of 500 cells/µl [21], but guidelines in Brazil and most countries recommend initiating treatment later [11].

Two significant changes in the AIDS definition in Rio de Janeiro occurred during the study period. First, in 1995, the definition was modified to include as an AIDS diagnosis any death certificate that mentioned AIDS as a basic cause of death. This was not previously done and is likely the reason for increased incidence of AIDS/tuberculosis co-infection between 1995 and 1996. Many AIDS patients die from tuberculosis and prior to 1996, a co-infected patient who died, but had not been reported to the Health Department as AIDS would have been counted only as a co-infection death, but not an AIDS case. This measure aimed to increase coverage of the surveillance system and in fact increased the amount of AIDS incidence in Brazil.

A second significant change in the AIDS definition in Rio de Janeiro occurred during the study period in 1998. At this time patients who were HIV infected and had a CD4 count <350 were considered to have AIDS. The number of AIDS cases peaked in 1998 in response to the definition change. If the change in definition had not occurred, it is likely that proportion trends would have stabilized beginning in 1997, rather than 1998. The potential effectiveness of ART in reducing tuberculosis incidence appears to have been relatively unfazed by this definition change. However, the proportion of AIDS patients diagnosed with tuberculosis within one year of AIDS diagnosis has been relatively stable for quite some time. Thus, it is possible that there is a limit to the effectiveness of antiretroviral therapy in reducing tuberculosis incidence at the population level.

The proportion of AIDS patients diagnosed with tuberculosis dropped substantially during the first several years of the study period, while the number of coinfected cases decreased markedly throughout the entire study period. Thus, it is likely that combined ART use influenced the overall decrease in tuberculosis incidence during the study period, or, at the very least helped prevent steep increases in tuberculosis incidence that would have otherwise occurred. AIDS-related tuberculosis in Rio de Janeiro decreased during the study period, whereas it increased in other countries with high rates of co-infection where ART was not available [1]. The observed stability of the proportion of AIDS patients developing tuberculosis since 1998 suggests that while combination ART should be considered an essential component of the response to the HIV and HIV / tuberculosis epidemics, it may not be sufficient alone to control tuberculosis among HIV-infected populations. Our results suggest that more specific interventions will be required if HIV-related tuberculosis incidence is to continue to decline.

A limitation in this study is that the linkage was conducted with the AIDS registry rather than an HIV registry because HIV is not a reportable infection in Brazil. Even though our analysis may underestimate the true burden of co-infection among HIV-infected patients, similar trends in co-infection are observed when we included tuberculosis cases diagnosed between one and three years prior to AIDS diagnosis (data not shown). It is likely that tuberculosis diagnosed within 1 year, and possibly up to three years, prior to an AIDS diagnosis represents HIV-TB co-infection. Thus, even though inclusion in our analysis relies on an AIDS diagnosis, it is likely that our data closely resemble HIV-TB co-infection.

A second limitation is the ecologic approach of investigating the association between decreasing co-infection and increased usage of antiretroviral therapy, in which associations should be seen with caution, since this kind of approach lacks a causal chain of events and could lead to the so-called ecologic fallacy. We did not link individual patient data with antiretroviral data, thus conclusions based on these analyses are primarily based on observation of trends in disease and number of patients receiving ART. Even though we do not have direct measures of HAART coverage in Brazil, we believe that it is high, since by law the government is obligated to provide free medication to all patients eligible for treatment according to Brazilian guidelines. Antiretroviral therapy has been shown to effectively reduce risk of tuberculosis in HIV-infected patients in cohort studies [6], thus extrapolation to population level data would be expected to yield similar trends.

Our data suggest that antiretroviral therapy, when distributed to a large proportion of the HIV-infected population, reduces the incidence of tuberculosis. However, tuberculosis remains a common problem, with 15% of AIDS cases experiencing and episode of tuberculosis in the year following diagnosis, most within 30 days. Because tuberculosis is so often an early complication of HIV, many patients will not have been given ART prior to becoming ill with tuberculosis. Further interventions are necessary to significantly reduce the tuberculosis burden among HIV-infected persons. Treatment of latent tuberculosis infection among HIV-infected patients [20], [21] and active tuberculosis case finding are strategies for potentially reducing tuberculosis/HIV burden. These and other interventions should be assessed to determine their effects in controlling tuberculosis in settings where ART use is being expanded.
